# Decision-Making in Research Tasks with Sequential Testing

**DOI:** 10.1371/journal.pone.0004607

**Published:** 2009-02-25

**Authors:** Thomas Pfeiffer, David G. Rand, Anna Dreber

**Affiliations:** 1 Program for Evolutionary Dynamics, Harvard University, Cambridge, Massachusetts, United States of America; 2 Department of Systems Biology, Harvard University, Cambridge, Massachusetts, United States of America; 3 Department of Economics, Stockholm School of Economics, Stockholm, Sweden; Science Commons, United States of America

## Abstract

**Background:**

In a recent controversial essay, published by JPA Ioannidis in PLoS Medicine, it has been argued that in some research fields, most of the published findings are false. Based on theoretical reasoning it can be shown that small effect sizes, error-prone tests, low priors of the tested hypotheses and biases in the evaluation and publication of research findings increase the fraction of false positives. These findings raise concerns about the reliability of research. However, they are based on a very simple scenario of scientific research, where single tests are used to evaluate independent hypotheses.

**Methodology/Principal Findings:**

In this study, we present computer simulations and experimental approaches for analyzing more realistic scenarios. In these scenarios, research tasks are solved sequentially, i.e. subsequent tests can be chosen depending on previous results. We investigate simple sequential testing and scenarios where only a selected subset of results can be published and used for future rounds of test choice. Results from computer simulations indicate that for the tasks analyzed in this study, the fraction of false among the positive findings declines over several rounds of testing if the most informative tests are performed. Our experiments show that human subjects frequently perform the most informative tests, leading to a decline of false positives as expected from the simulations.

**Conclusions/Significance:**

For the research tasks studied here, findings tend to become more reliable over time. We also find that the performance in those experimental settings where not all performed tests could be published turned out to be surprisingly inefficient. Our results may help optimize existing procedures used in the practice of scientific research and provide guidance for the development of novel forms of scholarly communication.

## Introduction

The testing of scientific hypotheses is typically associated with two types of statistical errors. A test may give confirmation for a hypothesis that is actually false. This type of error is commonly referred to as type I error or ‘false positive’. The probability α of obtaining a positive result although the hypothesis is false relates to the significance level of a test. Conversely, a test may fail to confirm a true hypothesis. This type of error is referred to as type II error or ‘false negative’. The probability β of missing a true relation corresponds to the power of a test, 1-β. The probability that a hypothesis is true after a test result has been obtained, i.e. the posterior probability, does not only depend on the test statistics, but also on the probability of the hypothesis before the test, i.e. the prior probability. For example, a positive result on a very improbable hypothesis is likely a false positive, while a positive result on a more probable hypothesis is more likely to be true. For a given prior probability, test result and test statistics, the posterior probability of a hypothesis can be calculated using Bayes' Theorem [1], [2].

In a recent controversial essay by J.P.A. Ioannidis [3], it has been argued that at least in some research fields, most of the published findings are false. This is because findings tend to be evaluated by p-value rather then posterior probability, and because positive results are more likely to be published than negative results. Small effect sizes, error-prone tests, low priors of the tested hypothesis, and biases in the interpretation of research findings can lead to a large fraction of published false positives [3]–[5]. Moreover, competition has been argued to have a negative effect on the reliability of research, because the same hypotheses are tested independently by competing research groups. The more often a hypothesis is tested independently, the more likely a positive result is obtained and published even if the hypothesis is false [3]. These findings raise concerns about the reliability of published research in those fields of the life sciences that are characterized by low priors, error-prone tests, and considerable competition.

Scientific research is, however, typically more complex than accounted for by the approach outlined in Ioannidis' essay, where single tests are used to evaluate single hypotheses. Research programs involve larger sets of hypotheses that are evaluated by different tests and complementary technical approaches. In many research fields, evidence from several tests and experiments has to be combined in order to reach a conclusion about a hypothesis. In such situations, it is often advantageous to evaluate hypotheses in a step-by-step manner, choosing each test based on previous findings. Such sequential testing is typically more cost-efficient than parallel testing, because previous knowledge often allows one to design experiments in a more informative way.

Sequential testing gives rise to temporal dynamics in the reliability of research. These dynamics are additionally affected by the fact that in scientific research, not all results are published or receive equal attention. Competition for limited space in scientific journals implies that some findings are not published at all, or are published in journals with low visibility. Especially those studies that do not achieve formal statistical significance are less likely to be published because they are perceived as less valuable [6], [7]. For studying the reliability of published findings in such scenarios, the methods outlined in Ioannidis' essay must be extended. In this study we use computer simulations and experimental approaches to analyze the impact of statistical errors on research programs that include sequential testing. We investigate simple scenarios of sequential testing as well as scenarios where not all results can be published and used for subsequent rounds of testing. To study reliability of research in these scenarios, we use simple research tasks that can be investigated with computer simulations as well as experimental settings.

For our experiments, these research tasks are framed within the context of molecular biology. Our framing gives participants a concrete picture of what they are investigating, and avoids situations where they have prior expectations or preferences for the hypotheses under investigation. However, our findings are not specific to molecular biology and may be generalized across fields that engage in hypothesis testing. Suppose that three genes (A, B, and C) are known to interact in a linear biochemical pathway: The first gene activates the second, which in turn activates the third. However, the order of the sequence is unknown. The task is to identify the correct sequence. There are six possible pathways (ABC, ACB, BAC, BCA, CAB, and CBA) that form the set of possible hypotheses. Knowledge of the pathway can be characterized by six probabilities p(h_1_), … , p(h_6_) that are associated with these hypotheses. In order to increase their knowledge about the hypotheses, researchers can test whether a specific gene activates another, i.e. they can test whether A activates B, A activates C, etc. Thus there are six different tests (AB, AC, BA, BC, CA, and CB). Note that each test supports two of the hypothesis and each hypothesis is supported by two tests. A positive result on test AB, for example, supports the sequences ABC and CAB, while sequence ABC is supported by positive results on test AB and BC.

All of the tests are equally prone to type I and type II errors. We use α = 0.12 and β = 0.3 in all our computer simulations and experiments. These values are higher than the values of α<0.05 and β<0.2 that researchers traditionally aim to achieve in the life sciences. We use these error probabilities to ensure that in the experiments, participants are exposed to errors at a considerable frequency. After a test has been performed, the probabilities associated with the hypotheses can be updated according to Bayes' Theorem. The research task is to identify the correct sequence after a limited number of tests. We use seven rounds of testing in all our simulations and experiments.

In these scenarios, individuals can choose tests depending on results that have been obtained earlier. If, for example, the interaction AB is tested in the first round, and the result is positive, it is an efficient strategy to test in the next round either BC or CA. These tests are the most informative ones, because they distinguish between the two hypothesis supported by the first result (CAB and ABC). In scenarios where several tests can be performed but not all results can be used for subsequent test rounds, some of the results have to be selected for publication. This implies that different results have to be compared and evaluated. Thus in contrast to quantifying the informativity of a test, here the informativity of a result has to be determined. If, for example, an individual receives a positive result on test AB and a negative result on test BA, and can only publish one of these results, it might be best to choose the positive result on AB because this result is more informative. The informativity of tests and results can be formally quantified using methods from information theory; see [8] for a review. Details about the informativity measures used in this study are given in the Methods section.

We perform computer simulations for three scenarios of sequential testing. First, we analyze a scenario of random test choice (SIM-R). Here, results from previous rounds are not used for the choice of a test. Second, we study a simple scenario with informative test choice (SIM-1). Third, we study a scenario of informative test choice where only a subset of results can be used for further test choice (SIM-2). To test predictions from the simulated scenarios with informative test choice, we use four different experimental settings. Two of these settings (EXP-1S and EXP-1G) are analogous to the simple scenario (SIM-1). The two other settings (EXP-2G and EXP-2E) are analogous to the complex scenario (SIM-2).

## Results

### Computer simulations

For the scenario with random test choice (SIM-R), in each round one of the 6 tests is chosen randomly. The result is sampled based on the error probabilities given above and is used to update the priors. In the first round, priors are 1/6 for all hypotheses. This is repeated for seven rounds. For random test choice, the reliability of published research follows the predictions from Ioannidis' approach for testing hypotheses with a single test. Given that two out of six tests support the true hypothesis, the frequency of false positives among the positive findings is given by: 2/3 α / (1/3 (1−β)+2/3 α)≈0.26, and stays constant over the rounds. The fraction of false negatives among the negative findings is 1/3 β / (1/3 β+2/3 (1−α))≈0.15.

**Figure 1:**
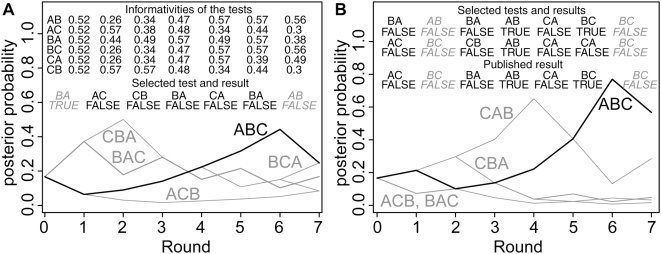
Sample Simulations. (A) Example simulation for the simple scenario with informative test choice (SIM-1). The correct sequence is ABC. In the first round, all hypotheses have the same prior probability of 1/6, and all tests have the same informativity. One test, BA, is chosen randomly and yields a positive. Since BA is not part of sequence ABC, this is a false positive. The probabilities for ABC and three other sequences decline, while the probabilities for the two sequences that contain BA (BAC and CBA) increase. In the next round, the tests AC and CB are the most informative ones. They distinguish between the two most likely hypotheses, BAC and CBA. AC is chosen and yields a negative result (true negative). This weakens hypothesis BAC and supports CBA. In the third round, CB is the most informative test. A negative result is obtained, and CBA and BAC are on par again. Further tests are performed, and yield correct answers, which establishes the correct sequence ABC as the most likely one. However, in the last round AB is tested and yields a false negative. The probability for ABC declines and finishes on par with BCA. (B) Example simulation for the complex scenario where in each round, two tests are performed but only one test results can be published. Again, ABC is the correct sequence. In first round, where all tests have the same informativity, two tests are chosen randomly. Both tests BA and AC yield a negative result and turn out to be equally valuable. The negative result on AC is randomly chosen to be published. This decreases the probabilities for ACB and BAC, and increases the probability for the four other sequences. In the second round, AB and BC are tested. Both tests yield false negatives, one of which (BC) is published. This leads to a decline for the probabilities of ABC and BCA. After a few rounds of testing, CAB is leading while the correct hypothesis ABC is second best. However, a true negative on CA bring CAB and ABC on par, and further tests establish ABC as the most likely sequence. In both panels, italic type codes for false positives and negatives.

In the simple scenario with informative test choice (SIM-1), previous test results are used for choosing a test: In each round, the priors associated with the hypotheses are calculated from previous results. Based on the priors, the informativity of each test is calculated (see Methods Section). The most informative test is selected. If there are several tests that have the highest expected informativity, one of them is chosen randomly. An example simulation for informative test choice is shown in Fig. 1A.

**Figure 2:**
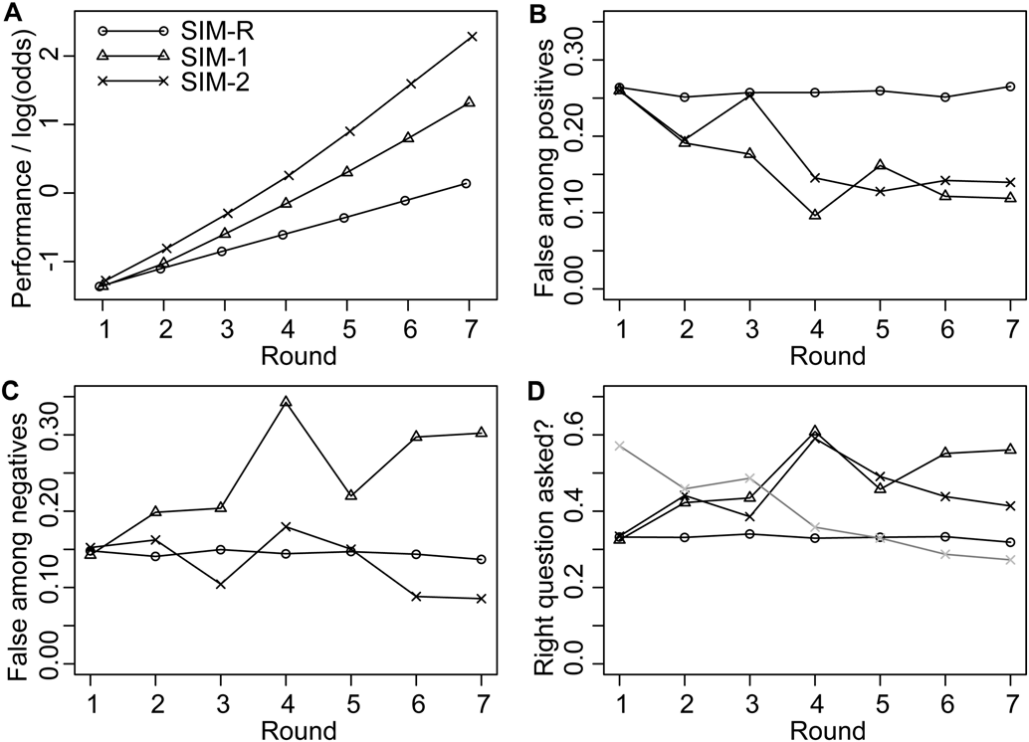
Simulation Results. (A) Evolution of knowledge. The odds for the true hypothesis increase at the slowest rate for random test choice (SIM-R), at intermediate rate for the scenario where the most informative test is chosen and published in each round (SIM-1), and at the fastest rate for the scenario where two tests are chosen in each round and the most informative test result is published (SIM-2). This illustrates that informative test choice leads to better performance than random test choice (SIM-1>SIM-R), and that there is an advantage of performing two tests even if only one test can be published (SIM-2>SIM-1). (B) Fraction of false among the positive results. For random test choice, the fraction of false positives stays constant at a level of 0.26. For both scenarios with informative test choice (SIM-1 and SIM-2), the fraction of false among the positives declines over the rounds. (C) Fraction of false among the negative results. For random test choice, the fraction of false among the negative results remains constant at a level of 0.15. For SIM-1 the fraction of false negatives tends to increase over the rounds, while for SIM-2 the fraction fluctuates around the level for random test choice. (D) Frequency of tests that support the true hypothesis. For random test choice, the chance of picking a test that is expected to support the true hypothesis (i.e. AB and BC for sequence ABC) is 1/3, because each hypothesis is supported by two of the six tests. Over the rounds, tests that support the true hypothesis tend to be chosen preferentially in the scenarios with informative test choice. This leads to a decrease of false among the positive findings. For scenario SIM-1, where all tests are published, this implies that there is an increase in the fraction of false negatives as shown panel C. For SIM-2, where results can be selected for publication, accumulating knowledge can be used to avoid the publication of false findings. The grey line shows the probability for a false finding to be published in SIM-2. The chance for a false finding to be published declines over the rounds.

For the more complex scenario of informative test choice (SIM-2), we assume that in each round two tests can be performed, but only one result can published, i.e. used in subsequent rounds. The two tests are selected independently of each other. First, for each test the expected informativity is calculated. Among the tests with the highest expected informativity, two are sampled randomly with replacement. This implies that if there is a single test that has the highest expected informativity, this test is performed twice. After the test results are obtained, the result with the highest informativity is published, while the other one is discarded. If both results are equally informative, one is chosen randomly. Details on the informativity of a result are given in the Methods section. An example simulation for this scenario is shown in Fig. 1B. For each of the three scenarios (SIM-R, SIM-1, SIM-2) we performed 10,000 simulations. Results are shown in Fig. 2.

As expected, performance is better in the scenarios with informative test choice than in the scenario with random test choice (Fig. 2A). The probability associated with the true hypothesis increases faster for informative test choice. Furthermore, performance is best for scenario SIM-2, where in each round two tests can be performed but only one can be published. Thus, although only one test is published per round, there is a clear advantage in having the opportunity to perform two tests and then choose the more informative result for publication.

Interestingly, for the scenarios with informative test choice (SIM-1 and SIM-2) the frequency of true positives shows a distinctive pattern. The fraction of false positives among the published positives declines over the rounds (Fig. 2B). For random test choice, the fraction remains constant at the level predicted by Ioannidis' approach. Thus, for the scenarios with informative test choice the estimate derived from Ioannidis' approach applies to the first round. In the long run, however, the fraction of false positives among the published findings tends to decrease. The fraction of false negatives among the published results increases in setting SIM-1, and remains approximately constant in setting SIM-2 (Fig. 2C).

What are the mechanisms behind these reliability patterns? Since in SIM-1 every result is published, a decrease in false positives can only result from an increased frequency of tests that support the correct sequence (such as AB and BC for sequence ABC). These tests are chosen because they tend to become more informative (Fig. 2D). This implies, however, that in SIM-1 the fraction of false negatives increases over the rounds (Fig. 2C). For the more complex scenario (SIM-2), there are two mechanisms that can contribute to a decrease in the fraction of false positives. The first mechanism is analogous to the mechanism driving the decrease of false positives in SIM-1. More tests tend to be performed that support the true hypotheses, because these tests are more informative (Fig. 2D). The second mechanism results from selecting one of the two tests for publication. Once knowledge about the hypotheses accumulates, it can be used to evaluate the reliability of the test results. Thus, as shown in Fig. 2D, publication of false findings can be avoided.

### Experimental design

To test the predictions from the computer simulations, we use four different experimental settings to study human performance. Research tasks in the experiments are analogous to the ones studied in the simulations. We focus specifically on the performance of the participants in comparison to the computer simulations, and whether their behavior leads to the predicted reliability patterns. Details about recruitment and participants are given in the Methods section.

In the first setting, participants solve single tasks. In each round, each participant chooses one test and obtains a test result. After 7 rounds of test choice, each participant is asked to determine the correct sequence. Participants earn $6 for each correct sequence and $2 for each incorrect sequence. We refer to this setting as EXP-1S, because single participants solve each task by choosing one test in each round.

In the second setting, participants interact in groups of 8 members to solve 8 tasks simultaneously. Each participant is involved exactly once in each of the 8 tasks. In each round, each participant receives the results of all previous tests on a specific task he/she has not contributed to yet. In the first round, this list is empty. The participants then choose a single test and obtain the result. The result is added to the list of previous results. When the next round starts, each participant passes her/his updated list to the next participant, and at the same time receives an updated list for a different task from another participant. After 7 rounds each participant must guess the correct sequence for the one task he/she has not contributed to yet. For each sequence that is identified correctly, all members of the group receive $1. As participants solve tasks in groups, we refer to this setting as EXP-1G.

Compared to EXP-1S, no differences in the dynamics of information gain can be expected. If individuals behave optimally, it does not matter whether the same or different participants performed previous tests. Nevertheless, comparison of the two settings helps to identify setting-specific factors such as the presence of under-confidence towards results of other participants, or other potential problems with the higher complexity of setting EXP-1G.

In the third setting, individuals interact in groups of 8 members. As in setting EXP-1G, they solve 8 tasks simultaneously, and each participant is involved exactly once in each of the tasks. However, in this setting, after receiving the list of previous results, individuals choose two tests. They receive both corresponding results, but only one of the two can be made available for the other participants. The other result has to be discarded. After receiving the results, each participant has to decide which of the two results they will add to the list of previous results. We refer to this setting as EXP-2G. As in the other settings, after 7 rounds each participant has to identify the correct sequence for the one task she/he has not contributed to yet. As in setting EXP-1G, for each correctly identified sequence all members of the group receive $1.

The fourth setting is similar to setting EXP-2G, but introduces independent test choice. While in each round of EXP-2G, each single participant chooses two tests, we now design a setting such that these two tests are chosen independently. To achieve this, there are three participants for each individual participant in EXP-2G. Two of them are assigned to the role of independent researchers while the third is assigned to the role of an editor. After receiving the results from previous rounds of testing, the two researchers independently choose one test each. They communicate their test results to the editor, who then chooses which of the two results to publish. Only this result is made available to the other researchers and editors in subsequent rounds. Because of the presence of editors, we refer to this setting as EXP-2E. It is analogous to scenario SIM-2. In total, 24 participants (8 triplets of two researchers and one editor) simultaneously solve 16 tasks, and receive $1 for each correct answer.

Moreover, we investigate whether knowing the error rates influences behavior in the experiments. Knowing the error rates is essential for determining the most informative test. However, given the complexity of the calculations required for determining informativity, it is likely that the participants use more simple heuristics. By not giving participants the error rates we can determine whether knowing this information influences test choice. In the four settings described above, participants were informed about the error rates. We investigate two additional settings that are identical to setting EXP-1S and EXP-2G, except that the participants were only informed about the potential presence of errors but not about the actual error rates. These settings are referred to as EXP-1S* and EXP-2G*, respectively.

### Experimental results

The correct sequence was identified in 283 of 440 tasks (64%). For the simple settings, the solution was correct for 60% (59/99) of the tasks in EXP-1S, 67% (59/88) in EXP-1G, and 70% (40/57) in EXP-1S*. This ranking is unexpected. One might have expected performance in EXP-1S to be better than in EXP-1S*, because in EXP-1S* participants do not know the error rates; and better than in EXP-1G, because the group setting might be more complex and confusing for the participants. For the more complex settings with selective publishing of results, the correct solution was identified in 65% of the tasks (68/104) in setting EXP-2G, 67% (32/48) in EXP-2G*, and 57% (25/44) in EXP-2E. This suggests that performance was worst in the setting with independent testing, and about equal in settings EXP-2G and EXP-2G*. A more detailed statistical analysis of performance is presented further below. To increase sensitivity, we use the probabilities associated with the true hypothesis after the last round of testing rather than the fraction of correct answers. For comparing performance of two settings we use two-sided t-tests on log-odd transformed probabilities. For comparing error frequencies in published results, we use two-sided Fisher's Exact Tests on the total numbers of true and false positives and negatives over all rounds. A summary of the results is given in Table 1.

**Table 1:** Results from the experiments and computer simulations.

*Setting*	*Number of tasks*	*Correctly solved*	*Performance: mean log odds, SE of mean*		*True Pos.*	*True Neg.*	*False Pos.*	*False Neg.*	*False among Pos.*	*False among Neg.*
*EXP-1S*	99	59	0.42	0.20	191	353	59	90	24%	20%
*EXP-1G*	88	59	0.86	0.22	196	310	34	76	15%	20%
EXP-1G*	57	40	0.81	0.25	128	196	28	47	18%	19%
***pooled***					**515**	**859**	**121**	**213**	**19%**	**20%**
*EXP-2G*	104	68	0.93	0.18	241	346	52	89	18%	20%
EXP-2G*	32	48	1.6	0.33	152	114	24	46	14%	29%
*EXP-2E*	25	44	0.11	0.34	90	140	33	45	27%	24%
***pooled***					**483**	**600**	**109**	**180**	**18%**	**23%**
*SIM-R*	10000	N.A.	0.14	0.02	16221	41195	5639	6945	26%	14%
*SIM-1*	10000	N.A.	1.3	0.02	23567	32115	4287	10031	15%	24%
*SIM-2*	10000	N.A.	2.3	0.03	29594	29472	4336	6598	13%	18%

**Figure 3:**
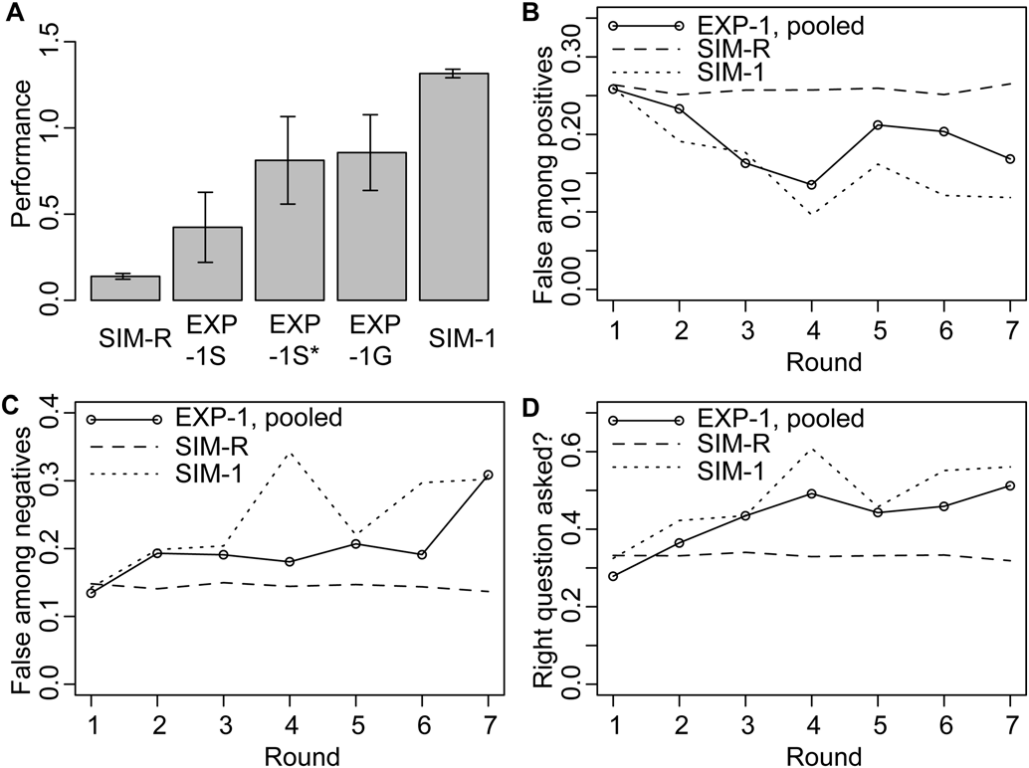
Experimental results for simple scenario where all results are published, and results from the corresponding simulations (SIM-R and SIM-1). (A) Performance (mean log odds for the true hypothesis after the last round, and standard error of the mean). Performance falls in between the performance for random test choice (SIM-R) and the simulated scenario with informative test choice (SIM-1). This indicates that informative tests tend to be chosen preferentially but not always. The performance in EXP-1S seems to be worse in EXP-1S* and EXP-1G. This implies that solving a task in the more complex group setting EXP-1G has no negative impact on performance. Moreover knowing the error rates seems not to be of advantage for problem solving in the experiment. (B) Fraction of false among the positive results. Data from all three simple settings are pooled for panels B–D. The dynamics of false positives follows the patterns expected from simulation SIM-1. Yet, it is less pronounced because the participants sometimes fail to select the most informative tests. (C) Fraction of false among the negative results. The pattern is as expected from simulation SIM-1. However, it is less pronounced because the participants sometimes fail to select the most informative test. (D) Frequency of those among the chosen tests that support the true hypotheses. Over the rounds, participants more often select those tests that correspond to the correct sequence. Thus false positives decrease while false negatives increase.

Figure 3 shows that for the simple scenarios with single tests in each round (EXP-1S, EXP-1S* and EXP-1G), the odds for the true hypotheses lie between the odds from the simulations with random test choice (SIM-R) and the simulations with informed test choice (SIM-1). Performance is better than for random test choice (t = 1.4, p = 0.17; t = 3.3, p = 0.002; t = 2.6, p = 0.01 for EXP-1S, EXP-1G, EXP-1S* vs. SIM-R), but not as good as for informed test choice (t = −4.4, p = 3e-5; t = −2.1, p = 0.04; t = −2.0, p = 0.05 for EXP-1S, EXP-1G, EXP-1S* vs. SIM-1). This implies that the participants preferentially choose informative tests, but sometimes failed to pick the most informative one. As indicated above, the performance in EXP-1G and EXP-1S* tends to be better than the performance in setting EXP-1S (t = 1.4, p = 0.15; t = 1.2, p = 0.23; for EXP-1G, EXP-1S* vs. EXP-1S). Thus, participants had no problems with the somewhat more complicated setting of solving several tasks simultaneously in groups (EXP-1G) rather than individually (EXP-1S). The observation that performance in EXP-1S* is better than performance in EXP-1S suggests that the participants could not take advantage of knowing the error rates. On the contrary, knowing the error rates seems to have a negative effect on the heuristics used for solving the tasks.

The patterns of published true and false positives roughly follow the predictions from the simulations. True positives increase over time while false positives stay constant, leading to a decreasing fraction of false among the positive findings (26% for SIM-R vs. 19% for pooled data from EXP-1S, EXP-1G and EXP-1S*; p = 9e-5). This decrease is less pronounced than in the simulations because the most informative test was not always selected (15% for SIM-1 vs. 19% for pooled data from EXP-1S, EXP-1G and EXP-1S*; p = 0.01).

**Figure 4:**
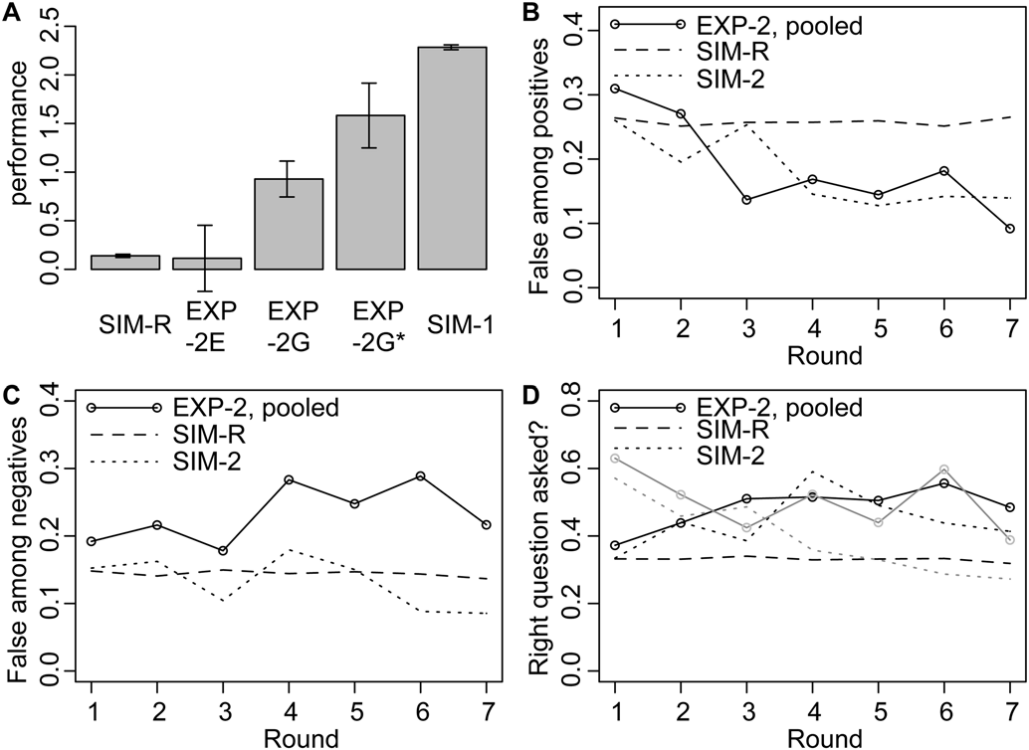
Experimental results for complex scenarios where in each rounds, two tests can be performed but only one result can be published. (A) Performance (mean log odds for the true hypothesis after the last round, and standard error of the mean). Performance for the settings with coordinated test choice (EXP-2G and EXP-2G*) falls in between the performance of simulations with random test choice (SIM-R), and informative test choice (SIM-2). Performance in the setting with independent test choice is worst, and not better than random. This suggests that there are substantial negative effects arising from independent testing. Analogous to EXP-1S and EXP-1S*, there seems to be a disadvantage for knowing the error rates: Participants in EXP-2G* outperform those in EXP-2G. (B) Fraction of false among the positive results. Data from all three complex settings are pooled for panels B–D. The dynamics of false positives follows the patterns expected from simulation SIM-2. (C) Fraction of false among the negative results. The fraction of false negatives in the experiments (EXP-2G, EXP-2G* and EXP-2E) is larger than expected from simulation SIM-2. (D) Frequency of those among the chosen tests that support the true hypotheses. Over the rounds, participants frequently select those tests that correspond to the correct sequence. This leads to a decline of false positives. In contrast to the simulations (SIM-2; dashed grey line), selection against false findings does not work efficiently (solid grey line). While in the simulations, false findings are increasingly selected against, there is no substantial improvement in avoiding publication of false findings in the experiment. This suggests that human subjects did not efficiently use background knowledge to avoid the publication of false findings.

For the more complex scenarios, we observe that the performance in EXP-2G and EXP-2G* falls in between the performance from the simulated scenarios with random test choice and the simulated scenario with informative test choice and subsequent selection (Fig. 4A; t = 4.3, p = 4e-5; t = 4.3, p = 8e-5; for EXP-2G, EXP-2G* vs. SIM-R; and t = −7.3, p = 6e-11; t = −2.1, p = 0.04; for EXP-2G, EXP-2G* vs. SIM-2). Surprisingly, performance in EXP-2G is not much better than in EXP-1G (t = −0.23; p = 0.8). Thus, the participants did not take advantage of the additional information they got from performing two tests, although the computer simulations clearly demonstrate that this is possible. Analogous to the differences between EXP-1S* and EXP-1S, the performance tends to be better in EXP-2G* than in EXP-2G (t = 1.7, p = 0.09). Again, we observe that informing participants about the error rates seems to have a negative impact on performance. Performance is worst in scenario EXP-2E (t = −2.1, p = 0.04 for EXP-2E vs. EXP-2G). In this scenario, performance is not better than random test choice (t = −0.07; p = 0.94). This outcome is surprising. One could expect that performance in EXP-2G is better than performance in EXP-2E, because in EXP-2G the participant can choose tests in a coordinated fashion, while in EXP-2E tests are chosen independently. However, that performance in EXP-2E is not better than random test choice indicates that in the experiments there is a substantial negative effect arising from independent test choice.

Publication patterns for EXP-2G, EXP-2G* and EXP-2E are similar to what is expected from the simulations. The frequency of false among positive findings decreases over time (Fig. 4B, 26% for SIM-R vs. 18% for pooled data from EXP-2G, EXP-2G* and EXP-2E; p = 3e-5), although this decrease is less pronounced then in the simulations with informative selection of tests and results (12% for SIM-2 vs. 18%; p = 1e-4). However, the frequency of false among negative findings (Fig. 4C) is higher than expected from the simulations with random test choice (14% for SIM-R vs. 23%; p = 1e-10), and the simulations with informative selection of tests and results (18% for SIM-2 vs. 23%; p = 0.0009). Over the rounds, participants increasingly chose tests that correspond to the true hypotheses, which explains the decrease in false positives (Fig. 4D). However, in contrast to the simulations (SIM-2) they fail to filter out false findings in the selection step (Fig. 4D). Thus, when choosing which test to publish, background knowledge from previous rounds of testing was not used efficiently.

## Discussion

Sequential testing and the use of previously obtained knowledge are essential characteristics of realistic research programs. In our study we extended previous simple approaches [3]–[5] to study reliability in research scenarios with sequential testing. We use computer simulations to derive predictions for the temporal patterns of reliability in these research scenarios. We then test these predictions using lab experiments on human decision making.

Our computer simulations indicate that for the tasks studied here, results tend to become more reliable over time if informative tests are performed. Previous approaches [3]–[5] do not explicitly capture this effect. They are therefore particularly suited to study the reliability of published research at the beginning of a research program, when little background knowledge is available and priors of the tested hypotheses are low. However, previous recommendations for improving the reliability of research [3], [5], [9] clearly apply to our scenarios as well.

An increase in the reliability of published research over time is in line with common intuition. At the beginning of a research program little is known and one would intuitively expect more false findings in the literature. Yet as research progresses, knowledge accumulates. A few competing hypotheses are developed, and are addressed more and more specifically. This leads to the testing of hypotheses with increasing prior probabilities, which in turn leads to a decreasing fraction of false positives among the positive findings in the literature.

Observations from the practice of scientific research support this scenario. In early stages of research there are often strong and contradictory claims. Many of these early claims eventually turn out to be wrong [10], [11], although this effect might not necessarily be driven solely by statistical errors. It could be argued that in research scenarios where not all findings can be published, an initial preference for extreme findings might not be irrational. This is because extreme findings tend to be more informative. In functioning research programs, knowledge should eventually converge towards a reliable consensus. However, early extreme findings might become problematic if they receive a disproportionate share of attention compared to later findings that refute the initial claim. This seems at least occasionally to be the case [12].

Unfortunately, it is difficult to get more detailed data for a quantitative analysis of the dynamics of reliability in research. To support our findings from the computer simulations, we therefore use lab experiments. Although such experiments do not replace an analysis of the practice of science, they can help identify factors that influence the reliability of scientific reasoning. Experiments on many aspects of human decision-making in the context of scientific research have been performed by psychologists [8], [13]–[17]. However, unlike in many of these experiments, here we do not focus on the heuristics and strategies used by humans in research. We mainly focus on the impact of the setting (i.e. sequential testing, testing with publication of selected results, and independent vs. coordinated test choice) on the performance of human subjects, and on the consequences for the reliability of published research.

Our experimental results roughly follow the patterns predicted by the simulations. The reliability of research increases over the rounds of testing. The increase is less pronounced than in the simulations, because human subjects did not always choose the most informative test. This is in agreement with the consensus in the psychological literature [17], indicating that human heuristics are well-adapted, but not necessarily optimal for a single, specific setting such as in our experiments. Moreover, our experiments indicate that in those settings where only a subset of findings can be used for further rounds of testing, performance is worse than the simulations predict. Additionally we find that independent rather than coordinated testing has a strong negative effect. This is in line with a call for more coordination rather than competition and independent testing in research [18].

Important aspects of scientific research are not reflected in our study. One phenomenon that may influence the outcome of our scenarios is herding behavior. Herding refers to a situation where individuals adopt the observed behavior or implied beliefs of other individuals. It has predominately been studied in the context of financial markets, where it can contribute to the formation of speculative bubbles [19], [20]. In the context of science, herding behavior occurs when numerous researchers perform similar experiments or interpret experimental results in a similar fashion. Several studies indicate that such behavior plays a role in scientific research [21], [22]. Because herding may lead to several groups independently performing the same set of experiments, it can amplify the negative effects of independent testing which are observed in our experiments.

Additionally, the incentive structure used in our experiments ensures that the interests of all participants are aligned with identifying the true hypothesis. This is not necessarily the case in scientific research, where editors and researchers may have conflicting incentives, or where competing researchers may follow different agendas. The impact of incentive structures on the performance of scientific research is an important issue which merits future theoretical and empirical studies.

Because of the inherent restrictions of laboratory approaches for studying human decision-making, we use very simple research tasks and scenarios to investigate the reliability of scientific results. We could not include important processes such as the development of tests or the emergence and formulation of hypotheses. We omitted these processes in order to focus solely on the impact of errors in a situation with well-defined hypotheses and tests with known error rates. It might be argued that tests with known error rates and hypotheses with well-defined prior probabilities hardly ever exist in real science. Because of the presence of systematic errors, error rates can be difficult to judge, and tend to be under-estimated by researchers [23]. Similarly, the prior probabilities of hypotheses are often subjective, and not as assessable as in our experiment. In the absence of well-defined probabilities of hypotheses it becomes impossible to quantify informativity.

Yet, assessing informativity is crucial for optimizing the allocation of resources such as time and money into experiments, and optimizing the publishing of experimental results. Therefore it is important to estimate prior probabilities. Modern information technology offers a number of mechanisms that may help researchers to efficiently “negotiate” their priors. Such mechanisms include Wikis, reputation systems and prediction markets [24]–[26]. We believe that a combination of theory and lab experiments can be helpful for investigating such novel mechanisms. While theory can help to optimize a novel implementation, experiments are essential to ensure that an implementation is not at odds with human heuristics and intuitions.

## Materials and Methods

### Bayesian Updating

The posterior probabilities after test *e_j_* are given by *p(h_i_|e_j_) = p(h_i_)p(e_j_|h_i_) / Σ_i_ p(h_i_)p(e_j_|h_i_)* for a positive test result, and *p(h_i_|∼e_j_) = p(h_i_)(1−p(e_j_|h_i_)) / Σ_i_ p(h_i_)(1−p(e_j_|h_i_))* for a negative test result. The probability *p(e_j_|h_i_)* of getting a positive result on test *j* given hypothesis *i* is true equals *1−β* if test *j* supports hypothesis *i*, and *α* if it does not support hypothesis *i*.

### Informativity of tests

A test is more informative if it is expected to change knowledge, i.e. the probabilities associated with the hypotheses. This can, for example, be quantified by the expected absolute changes of the probabilities (expected Manhattan distance between priors and posteriors probabilities [27]), or by the expected information gain (expected Kullback–Leibler divergence between priors and posteriors [28]). Although the optimal informativity measure may depend on the objectives of a research program and on what exactly the hypotheses are, different informativity measures typically yield very similar results [8]. For our simulations, we use the expected absolute changes of probabilities.

### Informativity of results

To compare the informativity of two results, we first calculate the posterior probabilities of the hypotheses using both results together. We next calculate the posterior probabilities using only result one, and only result two. Since only one of the two results can be published, we select the single result that brings us closest to the posterior after both results together. Thus, the result that minimizes the distance between the posterior after two results and the posterior after one result is chosen for publication, while the other test is discarded. As for the informativity of a test, we use absolute differences (Manhattan distance) as a distance measure.

### Experiments

In total, 212 participants were recruited by the CLER-Lab at Harvard Business School. Most participants were students from the Boston area. Median age was 21, and we had roughly equal numbers of male and female participants. Participants received a performance-independent show-up fee of $15 in addition to the payments earned in the experiments. The experiments were performed with 33 participants for setting EXP-1S, 19 participants for setting EXP-1S*, 4 groups of 8 participants for setting EXP-1G, 5 groups of 8 participants for setting EXP-2G, 2 groups of 8 participants for setting EXP-2G*, and 3 groups of 24 participants for setting EXP-2E. Participants in EXP-1S and EXP-1S* did 3 runs of problem solving. The participants in settings EXP-1G, EXP-2G, and EXP-2G* did either two or three runs of problem solving; in each of the runs the 8 members of a group solved 8 tasks simultaneously. Participants in setting EXP-2E did a single run of problem solving, where 16 tasks were solved simultaneously by 24 participants. In total, 440 tasks were solved (99, 57, 88, 104, 48, and 44 for EXP-1S, EXP-1S*, EXP-1G, EXP-2G, EXP-2G*, and EXP-2E respectively; four tasks in setting EXP-2E could not be used because participants failed to follow the instructions in these tasks). This involves almost 1,700 test choices in the settings EXP-1S, EXP-1S* and EXP-1G, more than 2,700 test choices and 1,300 choices of what result to publish in setting EXP-2G, EXP-2G*, and EXP-2E. The experiments have been approved by Harvard University CUHS (F14796-104). Written informed consent was obtained from all participants.
